# Phytochemical Analysis, Antioxidant, and Antimicrobial Activities of *Ducrosia flabellifolia:* A Combined Experimental and Computational Approaches

**DOI:** 10.3390/antiox11112174

**Published:** 2022-11-02

**Authors:** Mejdi Snoussi, Iqrar Ahmad, Abdullah M. A. Aljohani, Harun Patel, Mohammad A. Abdulhakeem, Yasser S. Alhazmi, Bektas Tepe, Mohd Adnan, Arif J. Siddiqui, Cengiz Sarikurkcu, Badraoui Riadh, Vincenzo De Feo, Mousa Alreshidi, Emira Noumi

**Affiliations:** 1 Department of Biology, College of Science, University of Hail, Ha’il 2440, Saudi Arabia; 2 Laboratory of Genetics, Biodiversity and Valorization of Bio-Resources (LR11ES41), Higher Institute of Biotechnology of Monastir, University of Monastir, Avenue Tahar Haddad, BP74, Monastir 5000, Tunisia; 3 Division of Computer Aided Drug Design, Department of Pharmaceutical Chemistry, R. C. Patel Institute of Pharmaceutical Education and Research, Shirpur 425405, Maharashtra, India; 4 Department of Molecular Biology and Genetics, Faculty of Science and Literature, TR-79000 Kilis, Turkey; 5 Faculty of Pharmacy, Afyonkarahisar Health Sciences University, TR-03100 Afyonkarahisar, Turkey; 6 Section of Histology Cytology, Medicine Faculty of Tunis, University of Tunis El Manar, La Rabta 1007, Road Djebal Lakhdhar, Tunis 1007, Tunisia; 7 Department of HistoEmbryology and Cytogenetics, Medicine Faculty of Sfax, University of Sfax, Road of Majida Boulia, Sfax 3029, Tunisia; 8 Department of Pharmacy, University of Salerno, Via Giovanni Paolo II, 132, Fisciano, 84084 Salerno, Italy; 9 Molecular Diagnostics and Personalized Therapeutics Unit, University of Hail, Ha’il 2440, Saudi Arabia

**Keywords:** *Ducrosia flabellifolia*, chemical composition, antioxidant, antimicrobial, molecular docking, dynamic simulation

## Abstract

*Ducrosia flabellifolia* Boiss. is a rare desert plant known to be a promising source of bioactive compounds. In this paper, we report for the first time the phytochemical composition and biological activities of *D. flabellifolia* hydroalcoholic extract by using liquid chromatography–electrospray tandem mass spectrometry (ESI-MS/MS) technique. The results obtained showed the richness of the tested extract in phenols, tannins, and flavonoids. Twenty-three phytoconstituents were identified, represented mainly by chlorogenic acid, followed by ferulic acid, caffeic acid, and sinapic acid. The tested hydroalcoholic extract was able to inhibit the growth of all tested bacteria and yeast on agar Petri dishes at 3 mg/disc with mean growth inhibition zone ranging from 8.00 ± 0.00 mm for *Enterococcus cloacae* (*E. cloacae*) to 36.33 ± 0.58 mm for *Staphylococcus epidermidis*. Minimal inhibitory concentration ranged from 12.5 mg/mL to 200 mg/mL and the hydroalcoholic extract from *D. flabellifolia* exhibited a bacteriostatic and fungistatic character. In addition, *D. flabellifolia* hydroalcoholic extract possessed a good ability to scavenge different free radicals as compared to standard molecules. Molecular docking studies on the identified phyto-compounds in bacterial, fungal, and human peroxiredoxin 5 receptors were performed to corroborate the in vitro results, which revealed good binding profiles on the examined protein targets. A standard atomistic 100 ns dynamic simulation investigation was used to further evaluate the interaction stability of the promising phytocompounds, and the results showed conformational stability in the binding cavity. The obtained results highlighted the medicinal use of *D. flabellifolia* as source of bioactive compounds, as antioxidant, antibacterial, and antifungal agent.

## 1. Introduction

The Apiaceae family (syn. Umbelliferae) comprises more than 455 genera and more than 3700 species [1] and is known to yield distinctive phytochemicals with antioxidant, antimicrobial, anticancer, anti-inflammatory, and hepatoprotective properties [2,3,4]. In Saudi Arabia, the Apiaceae family comprises more than eighteen plant species and is considered the most used family in ethnomedicine [5,6]. The *Ducrosia* genus includes six species and is widely spread in Asia, particularly the Kingdom of Saudi Arabia, Afghanistan, Pakistan, and Iraq with *D. anethifolia* as the most popular species [7]. *Ducrosia flabellifolia* Boiss. (*D. flabellifolia*), with the popular name of “Al Haza”, grows as a rare species in volcanic cinders in the center and north of Saudi Arabia [8,9], and in the deserts of the eastern parts of Jordan [10].

Many scientific studies have investigated the chemical composition of *D. anethifolia* and *D. flabellifolia* essential oil from Saudi Arabia [11], Iran [12], Jordan [13,14,15], and Tunisia [16]. Most studies have focused on the essential oil obtained from *D. flabellifolia* species. In fact, in 2014, Shahabipour and colleagues [12] reported the identification of 32 bioactive compounds in the volatile oil of *D. flabellifolia* from Iran dominated mainly by decanal (32.8 ± 1.91%), dodecanal (32.6 ± 1.75), and (2E)-tridecen-1-al (3.3 ± 0.08%). Moreover, *D. flabellifolia* volatile oil from Safawi (Jordan) obtained by hydrodistillation was a rich source of monoterpenes and terpenoids [13]. Hydrodistilled oil from fresh leaves was dominated by n-decanal (36.61%), dodecanal (7.5%), D-L- limonene (3.86%), and β-phellandrene (3.84%), while the oil obtained by hydrodistillation from dried leaves was dominated by n-decanal (24.44%), α-pinene (15.72%, 2E-octene (9.73%), 2Z-octane (7.04%), 2-heptanone (5.92%), frenchone (5.18%), and β-phellandrene (4.58%) [13]. While using GC/MS technique, Elsharkawi and colleagues [11] reported the identification of 30 phytoconstituents in ethyl acetate fraction of *D. anethifolia* collected from Wadi Arar (Saudi Arabia) dominated by 8-ethoxypsoralen, coumarin-6-ol-3,4-dihydro-4,4,7,8-tetramethyl, isoaromadendrene epoxide, aromadendrene oxide, ferulic acid methyl ester, pterin-6-carboxylic acid, vitamin A palmitate, and ursodeoxycholic acid.

The pharmacological potency of organic extracts obtained from members of *Ducrosia* genus has been subjected to diverse in vitro and in vivo biological activities. In a former study performed by Javidnia et al. [17], potent antimicrobial activity of the hydro-methanolic extract from *D. anethifolia* aerial parts towards *Bacillus subtilis*, *Staphylococcus aureus*, *Escherichia coli*, *Pseudomonas aeruginosa,* and *Candida albicans* has been assessed. For a long time, the aerial parts and leaves of *D. flabellifolia* have been smoked as a cigarette [18]. The aerial parts of *D. ismaelis* have been reported as being used as natural insecticides and to cure skin infections [13,19]. In addition, *D. anethifolia* has been well documented essentially as an insecticide and is used for the treatment of colds [20], heartburn [21], inflammation of the inner wall of the nose [22], as an analgesic [23], and a flavoring in food [24,25].

The emergence of multidrug-resistant bacterial pathogens due to the overuse and abuse of currently available antibiotics has caused about 750,000 deaths annually, and 10 million will die every year by 2050 [26]. In this context, the purpose of this study was to ascertain the effective valorization of selected *D. flabellifolia* hydroalcoholic extract collected from the Hail region (Saudi Arabia) by assessing its phytochemical profile, antioxidant, and antimicrobial activities. Molecular docking and dynamic approaches were performed in order to elucidate the possible interaction between the identified phytoconstituents with specific target proteins involved in antibacterial, antifungal, and antioxidant activities.

## 2. Materials and Methods

### 2.1. Chemicals

Gallic acid, (+)-catechin, pyrocatechol, chlorogenic acid, 2,5-dihydroxybenzoic acid, 4-hydroxybenzoic acid, (−)-epicatechin, caffeic acid, syringic acid, vanillin, taxifolin, sinapic acid, p-coumaric acid, ferulic acid, rosmarinic acid, 2-hydroxycinnamic acid, pinoresinol, quercetin, luteolin, and apigenin were purchased from Sigma-Aldrich (St. Louis, MO, USA). Vanillic acid, 3-hydroxybenzoic acid, 3,4-dihydroxyphenylacetic acid, apigenin 7-glucoside, luteolin 7-glucoside, hesperidin, eriodictyol, and kaempferol were obtained from Fluka (St. Louis, MO, USA). Finally, verbascoside, protocatechuic acid, and hyperoside were purchased from HWI Analytik (Ruelzheim, RP, Germany). Methanol and formic acid of HPLC grade were purchased from Sigma-Aldrich (St. Louis, MO, USA) and Merck (Darmstadt, Hesse, Germany), respectively. Ultra-pure water (18 mΩ) was obtained using a Millipore Milli-Q Plus water treatment system (MILLIPORE CORPORATION, Bedford, MA, USA).

### 2.2. Plant Material Sampling

**Figure 1:**
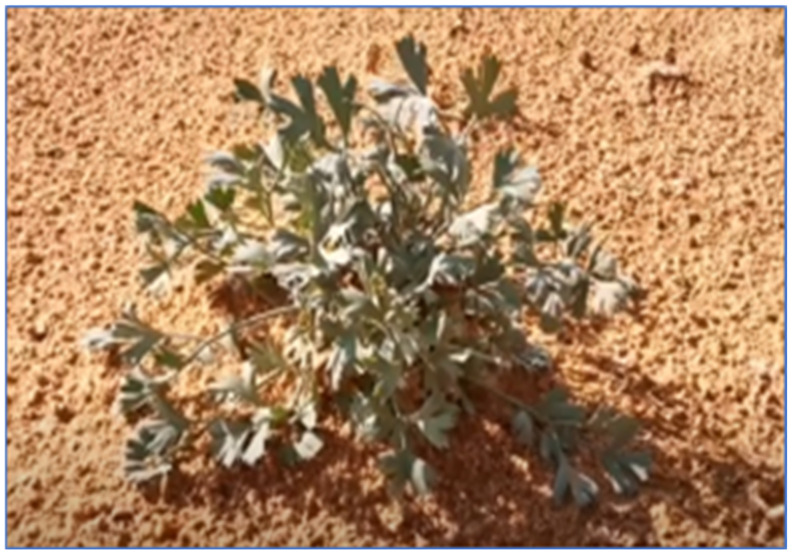
*D. flabellifolia* Boiss. plant species collected from the Hail region.

In this proposal, *D. flabellifolia* (Al-Hazaa; Figure 1) plant species were collected from the Hail region (Al-Mu’ayqilat, 27°16′41.9″ N, 41°22′48.0″ E) in October 2019. A voucher specimen (AN04) was deposited at the herbarium in the Department of Biology (College of Science, University of Hail, Hail, Kingdom of Saudi Arabia). For the experiment, 4 g of air-dried aerial parts were macerated in 100 mL of methanol-80% at room temperature for 72 h. The filtrate was recuperated by lyophilization, and the yield (expressed in percentage) was calculated using the following Equation (1):

Yield (%) = (W1/100)/W2, (1)

where W1 is the weight of extract after the evaporation of solvent and W2 is the dry weight of the sample. The yield of extraction was about 21.56 ± 1.78%.

### 2.3. Study of the Phytochemical Composition

An Agilent Technologies 1260 Infinity liquid chromatography system (Santa Clara, CA, USA) hyphenated to a 6420 Triple Quad mass spectrometer was used for quantitative analyses. Chromatographic separation was carried out on a Poroshell 120 EC-C18 (100 mm × 4.6 mm I.D., 2.7 μm) column (Santa Clara, CA, USA). The previously validated method was used for the analysis of phenolic compounds by LC-ESI-MS/MS [27]. The mobile phase was made up of solvent A (0.1%, *v/v* formic acid solution) and solvent B (methanol). The gradient profile was set as follows: 0.00 min 2% B eluent, 3.00 min 2% B eluent, 6.00 min 25% B eluent, 10.00 min 50% B eluent, 14.00 min 95% B eluent, 17.00 min 95% B, and 17.50 min 2% B eluent. The column temperature was maintained at 25 °C. The flow rate was 0.4 mL min^−1^ and the injection volume was 2.0 μL. The tandem mass spectrometer was interfaced with the LC system via an ESI source. The electrospray source of the MS was operated in negative and positive multiple reaction monitoring (MRM) mode and the interface conditions were as follows: capillary voltage of −3.5 kV, gas temperature of 300 °C, and gas flow of 11 L min^−1^. The nebulizer pressure was 40 psi. MRM transitions, the optimum collision energies, and retention times for each species are indicated in Supplementary Material S1. In addition, representative LC-ESI-MS/MS chromatograms of phenolic compounds are shown in Supplementary Material S2. Calibration curves and sensitivity properties of the method are also shown in Supplementary Material S3.

In negative and positive multiple reaction monitoring (MRM) mode, the peaks of the analytes were identified by comparing the retention time, together with monitoring ion pairs in an authentic standard solution.

### 2.4. Screening of the Biological Activities 

#### 2.4.1. Antimicrobial Activities

The antimicrobial activity of Al-Haza extracts was tested against twelve ESKAPE bacterial strains including *Enterococcus faecium*, three *Staphylococcus* species (*S. aureus*, *S. epidermidis*, and *S. hominis*), *Klebsiella pneumoniae*, *Acinetobacter baumannii*, *Pseudomonas aeruginosa*, two *Enterobacter species (E. cloacae* and *E. faecalis*), and *Escherichia coli*. Four types of *Candida* species were also tested (*C. albicans* ATCC 20402, *C. tropicalis* ATCC 1362. *C. guillermondii* ATCC 6260, and *C. utilis* ATCC 9255). The effect of the hydroalcoholic extract from *D. flabellifolia* was estimated using disc diffusion assay [28] by measuring the diameter of the growth inhibition zone tested on a Mueller Hinton agar plate for bacterial strains and Sabouraud dextrose agar for yeast. Sterile discs were impregnated with three different concentrations of the tested extract (1 mg/disc, 1.5 mg/disc, and 3 mg/disc). After incubation, the mean diameter of the growth inhibition zone (mGIZ) was calculated, and the scheme proposed by Parveen et al. [29] was used to interpret the obtained results (no activity: mGIZ = 0; low activity: mGIZ = 1–6 mm; moderate activity: mGIZ = 7–10 mm; high activity: mGIZ = 11–15 mm; and very high activity: GIZ = 16–20 mm). Ampicillin and Amphotericin B were used as controls. 

The microdilution assay was used for the determination of MIC (minimal inhibitory concentration) and MBC/MFC values (minimal bactericidal/fungicidal concentration) as previously described by Khalfaoui et al. [30]. Bacterial and fungal cultures were inoculated into the wells of 96-well microtiter plates in the presence of *D. flabellifolia* methanolic extract at final concentrations varying from 0.039 mg/mL to 100 mg/mL. To interpret the character of the tested extract, we used the ratios (MBC/MIC ratio and MFC/MIC ratio) described by Gatsing et al. [31] and Moroh et al. [32].

#### 2.4.2. Phytochemistry and Antioxidant Activities Screening

Total phenolic content expressed as milligram (mg) of gallic acid per gram of plant extract (mg GAE/g extract) was estimated by using the Folin–Ciocalteu method as previously described by Kumar et al. [33]. Total flavonoids expressed as mg of quercetin equivalents per gram of plant extract (mg QE/g extract) were determined using the AlCl_3_ method developed by Benariba et al. [34]. In addition, acidified vanillin method previously described by Broadhurst and Jones [35] was used to estimate the total condensed tannins (expressed mg tannic acid equivalent per gram of plant extract (mg TAE/g extract)). 

The ability of the Al-Haza extract against DPPH-H was determined following the same method as Mseddi al. [36]. The method of Koleva et al. [37] for β-Carotene bleaching test and Oyaizu [38] method for the determination of reducing power were used.

### 2.5. In Silico Study

#### 2.5.1. Molecular Docking

The 3D structures of LC-MS-detected phytochemicals were retrieved in structural data format (sdf) from the PubChem database. The LigPrep module was used to prepare the entire set of phytochemicals, which involved the addition of hydrogen atoms and suitable charges, as well as the correcting of the valences and the protonation and tautomeric states at pH 7.2 ± 2.0 of the molecules using the Epik tool [39,40]. The X-ray crystal structure of *S. aureus* type IIA topoisomerase, tyrosyl-tRNA synthetases (TyrRS) of *S. aureus*, Sap1 of *C. albicans*, and human peroxiredoxin 5 receptor were retrieved from the PDB server with accession codes of 2XCT, 1JIJ, 2QZW, and 1HD2, respectively, as the receptor for the molecular docking study. The Schrodinger Protein Preparation Wizard has been used for protein preparation and energy minimization, in which crystallographic water molecules are removed, then missing hydrogen and/or side chain atoms are added, and the correct charges and protonation states are given to protein residues at pH 7.0 [41,42]. The protein structure was energy minimized using the OPLS3 force field to alleviate steric conflicts in the protein structure. Following that, the prepared protein was considered for grid generation utilizing the “Receptor Grid Generation” panel where active sites in targeted proteins were defined for grid generation by selecting cognate ligands [43]. Glide’s standard precision (SP) mode was used to execute all docking calculations for the prepared ligand molecules, with the default parameters.

#### 2.5.2. Molecular Dynamics (MD) Simulation

MD simulation is regarded as the most important approach for comprehending the nature of biological macromolecules’ underlying structure and function. The MD simulations were conducted utilizing the Schrödinger MD simulation program Desmond, which helps us to understand how a ligand–protein complex binds in simulated physiological conditions [44]. MD simulation analysis was conducted for top score phyto-compound Hyperoside in complex with 1JIJ protein. The simulation system was built through the system builder, with the boundaries set by an orthorhombic shape with a diameter of 10 Å × 10 Å × 10 Å and filled with SPC water molecules. 

To neutralize the system’s charges, sodium (+27) and chloride (−12) ions were supplied as counter ions and salt concentration was maintained at 0.15 M. Following the setup of the system builder, the protein–ligand complex system was minimized using the steepest descent method, which was followed by LBFGS algorithms with a maximum of 2000 iterations [45,46]. Further, the system was equilibrated using the NPT ensemble with a Nose–Hoover chain thermostat to evenly distribute the ions and solvent throughout the protein–ligand complex. During the simulation, the temperature was maintained at 300 K. Furthermore, isotropic position scaling was used to control 1bar barostat pressure [47,48,49,50]. A 100 ns simulation was run, with a total of 1000 frames stored in the simulation system for subsequent analysis using the Desmond program’s “Simulation Interactions Diagram” module.

### 2.6. Statistical Analysis

All experiments were performed in triplicate and average values were calculated using the SPSS 25.0 statistical package for Windows. Duncan’s multiple-range tests for means with a 95% confidence interval (*p* ≤ 0.05) were used to calculate the differences in means. 

## 3. Results

### 3.1. Phytochemical Composition of D. Flabellifolia Hydroalcoholic Extract

Using ESI-MS/MS technique, 23 phytocompounds were identified in the hydroalcoholic extract from *D. flabellifolia* aerial parts (Table 1). This extract was dominated by (mg/g of extract): chlorogenic acid (5980.96 ± 73.12), ferulic acid (180.58 ± 2.77), caffeic acid (70.90 ± 1.75), and sinapic acid (61.74 ± 2.79).

**Figure 2:**
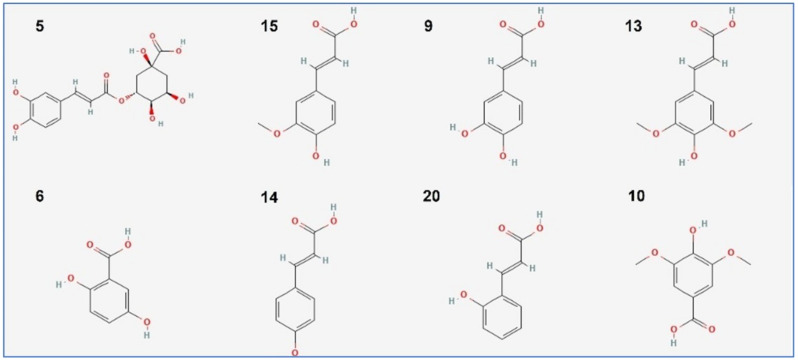
Chemical structure of the main compounds identified by ESI-MS/MS technique in the hydroalcoholic extract from *D. flabellifolia* aerial parts. Numbers are the same as listed in Table 1.

The chemical structures of the main identified phytoconstituents are summarized in Figure 2.

### 3.2. Antioxidant Activities Screening

Table 2 summarizes the results of the quantification of tannins, phenols, and flavonoids. In fact, the hydroalcoholic extract was dominated by phenolic compounds (46.684 ± 0.757 mg GAE/g extract), followed by tannins (6.204 ± 0.401 mg TAE/g extract), and flavonoids (1.816 ± 0.133 mg QE/g extract). The results obtained showed that the tested methanolic extract from *D. flabellifolia* was able to scavenge the DPPH radical with a low IC_50_ value (0.014 ± 0.045 mg/mL) as compared to BHT and AA (0.023 ± 3 × 10^−4^ mg/mL and 0.022 ± 53 × 10^-4^ mg/mL, respectively). In addition, the ABTS + radical was scavenged with an IC_50_ value of about 0.102 ± 0.024 mg/mL of the tested extract. For beta-carotene bleaching inhibiting property, the IC_50_ value was estimated at 7.80 ± 0.919 mg/mL, significantly different from BHT and AA (*p* < 0.05).

### 3.3. Antimicrobial Activities Screening

**Figure 3:**
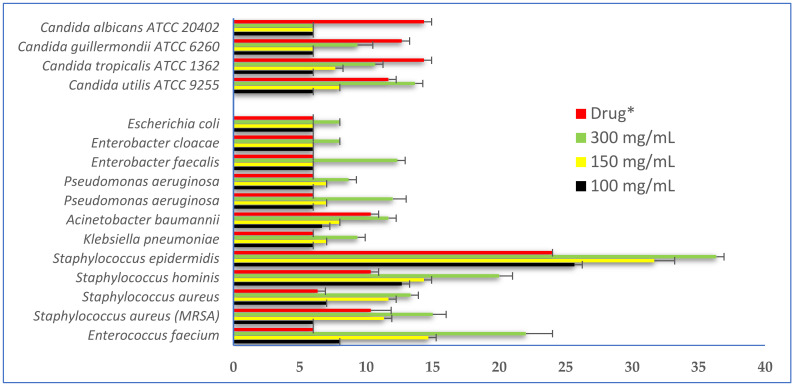
Mean diameters of bacterial and fungal growth inhibition zones (mGIZ ± mm) obtained with different concentrations of *D. flabellifolia* hydro-methanolic extract as compared to standard drugs. *: Ampicillin for bacteria and amphotericin B for *Candida* strains.

The results of the antimicrobial activity of the hydro-methanolic extract from *D. flabellifolia* aerial parts showed that the mean diameter of the growth inhibition zone (mGIZ) increased in a concentration-dependent manner (Figure 3). At 3 mg/disc, the mGIZ ranged from 8.00 ± 0.00 mm (*E. cloacae*) to 36.33 ± 0.58 mm (*S. epidermidis*). At the same concentration (3 mg/disc), *C. utilis* ATCC 9255 was the most sensitive strain with mGIZ of about 13.67 ± 0.58 mm. All bacterial strains seem to be more sensitive to the tested extract6 as compared to yeast strains, and the reference drug (ampicillin). Using the scheme proposed by Parveen et al. [29], the tested extract at 3 mg/mL showed high to very high activity against all tested bacterial and fungal strains with mGIZ from 11 to 20 mm.

Using the microdilution technique, the minimal inhibitory concentrations values (MICs) ranged from 12.5 to 25 mg/mL for bacterial strains and the minimal bactericidal concentration values (MBCs) ranged from 100 to 200 mg/mL. Using the scheme proposed by Gatsing et al. [31] and Moroh et al. [32], the tested extract showed bacteriostatic activity against almost all tested ESKAPE microorganisms (MBC/MIC ratio higher than 4 and lower than 16), with the exception of *E. faecium* and *E. cloacae* (MBC/MIC equal to 4). All these data are summarized in Table 3.

Similarly, the tested *D. flabellifolia* hydroalcoholic extract was able to inhibit the growth of *Candida* strains in liquid media at 25 mg/mL (Table 4), while a high concentration of the tested extract was needed to completely kill them (from 100 to 200 mg/mL), with MFC/MIC ratios varying from 4 (C. albicans ATCC 20402; fungicidal action) to 8 (Fungistatic action against *C. utilis* ATCC 9255, *C. tropicalis* ATCC 1362, and *C. guillermondii* ATCC 6260).

### 3.4. Computational Study

#### 3.4.1. Molecular Docking 

**Figure 4:**
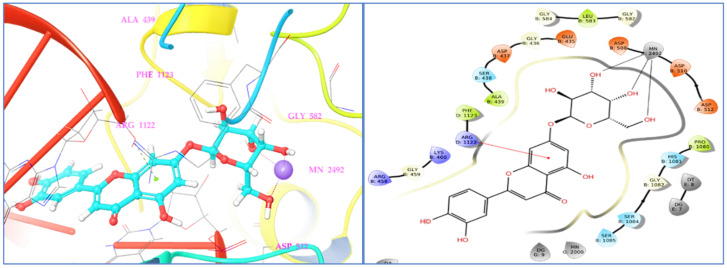
Two- and three-dimensional residual interactions network of luteolin 7-glucoside against the active site of *S. aureus* IIA topoisomerase (PDB ID: 2XCT).

The molecular docking study of identified phyto-compounds was carried out against bacterial fungal and human peroxiredoxin 5 targets. The binding affinities of phyto-compounds are shown in the Supplementary Material (S1). The results of the molecular docking study revealed that phyto-compound luteolin 7-glucoside showed the highest binding affinity toward topoisomerase IIA (2XCT) with a docking score −12.562 Kcal/mol. The phyto-compounds apigenin 7-glucoside, quercetin, (−)-epicatechin, eriodictyol, and hyperoside from *D. flabellifolia* also showed significant binding affinities against topoisomerase IIA (2XCT) with docking scores of −12.514, −10.06, −9.836, −9.582, and −9.159 Kcal/mol, respectively. The best binding pose of luteolin 7-glucoside inside the active site of topoisomerase IIA and the 2D and 3D representations of the interactions with the amino acids inside the binding pocket are presented in Figure 4.

**Figure 5:**
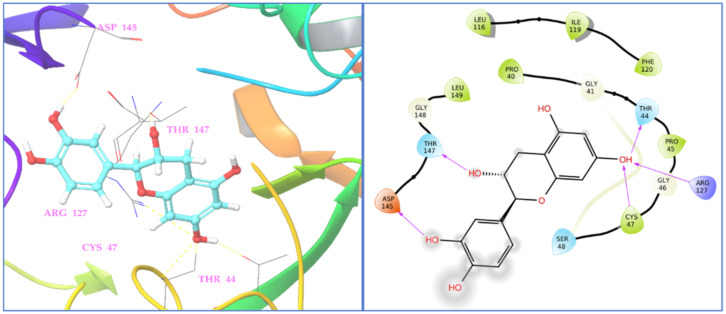
Two- and three-dimensional residual interactions network of (−)-epicatechin in human peroxiredoxin 5 protein (PDB ID: 1HD2).

Luteolin 7-glucoside binding interaction reveals that it interacted with Arg1122 and manganese (II) ion via π-cationic and metal coordinates, respectively. Among the assessed phyto-compounds, five compounds, namely, (−)-epicatechin, eriodictyol, 2,5-dihydroxybenzoic acid, chlorogenic acid, luteolin 7-glucoside, and vanillin showed proper and promising antioxidant activity by proper recognition at the binding active site of human peroxiredoxin protein with docking scores of −5.791, −5.255, −5.249, −5.11, −5.06, and −5.02 Kcal/mol, respectively. It was reported that hydrogen bonds and hydrophobic interactions established with the surrounding amino acids are applied to anticipate the binding modes of the novel compounds compared to the antioxidant benzoic acid cognate ligand, at the 1HD2 complex. This active pocket contained conserved amino acid residues such as Thr44, Gly46, Cys47, and Arg127, which play critical roles in docked compound recognition via hydrogen bonding and hydrophobic interactions. The binding interaction shows that two terminal hydroxyl groups connected with asp145 and the147 in the (−)-epicatechin-1HD2 complex, whereas the chromane ring hydroxyl group showed triple hydrogen bonding with Cys47, Thr44, and Arg127 amino acids, suggesting that the identified phyto-compound (−)-epicatechin has antioxidant activity (Figure 5).

**Figure 6:**
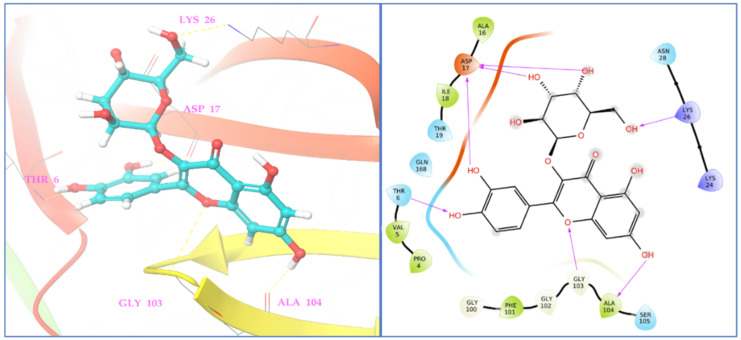
Two- and three-dimensional residual interactions network of hyperoside against the active site of *C. albicans* Sap1 (PDB ID: 2QZW).

Similarly, in *C. albicans* Sap1 protein (2QZW), hyperoside showed the highest binding affinity with a docking score of −6.055 Kcal/mol, and phyto-compounds eriodictyol, (−)-epicatechin, and syringic acid showed potential binding affinities against *C. albicans* Sap1 (2QZW) protein with docking scores of −5.814, −5.461, and −5.042 Kcal/mol, respectively. The residues of Thr6, Asp17, Lys26, Gly103, and Ala104, from Sap1 of *C. albicans* formed seven significant interactions with the phyto-compound hyperoside with a standard hydrogen binding pattern as shown in Figure 6.

**Figure 7:**
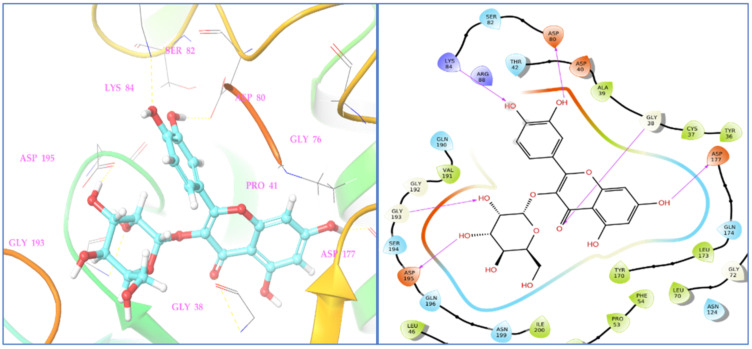
Two- and three-dimensional residual interactions network of hyperoside against the active site of *TyrRS* protein (PDB ID: 1JIJ).

Among assessed phyto-compounds, hyperoside also showed a promising docking score (−8.852 Kcal/mol) in the microbial target TyrRS from S. aureus (1JIJ) in which it interacted with active residues, namely, Asp195, Gly193, Gly38, and Asp177 (Figure 7). 

Table 5 shows the promising phyto-compounds hydrogen bonding profile in the active site of type IIA topoisomerase, TyrRS, Sap1, and human peroxiredoxin 5 proteins.

#### 3.4.2. MD Simulation 

**Figure 8:**
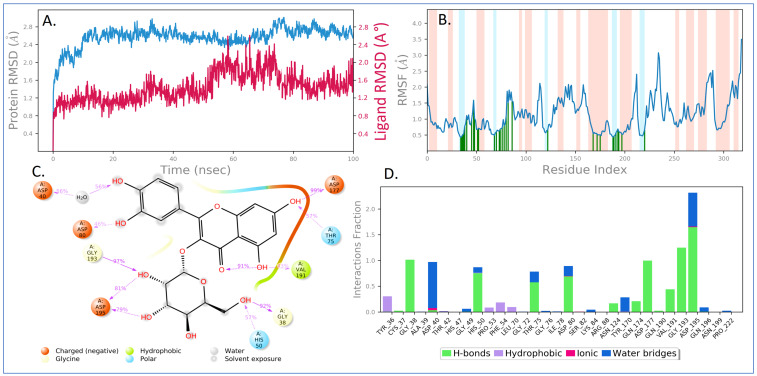
MD simulation analysis of hyperoside in complex with *S. aureus tyrosyl-tRNA synthetases* (*TyrRS*) (PDB ID: 1JIJ): (**A**) time dependent RMSD (protein Cα atoms RMSD is shown in teal blue color while the RMSDs of hyperoside with respect to protein are shown in brown color); (**B**) protein Cα atoms RMSF; (**C**) 2D diagram of ligand interactions that occurred more than 30.0% of the simulation time; and (**D**) protein–ligand contact analysis of throughout the simulation.

Using molecular dynamics simulation, the docked complex of the phyto-compound hyperoside at the binding site of TyrRS of *S. aureus* was simulated under biological environments to investigate complex stability and protein flexibility. Hyperoside, a phyto-compound, has shown a high affinity for microbial targets, hence it was chosen for the MD simulation studies. The MD trajectories were used to determine RMSD values, root-mean-square fluctuation (RMSF) values, and protein–ligand interactions. Various MD trajectory data analyses for the hyperoside-1JIJ complex are shown in Figure 8. The RMSD figure showed a stable ligand–protein complex throughout the simulation time, as shown by RMSD values ranging from 1.6 to 2.8 Å for protein Cα atoms in the complex with hyperoside (Figure 8A). RMSD changes remain within 3 Å throughout the simulation period, which is perfectly appropriate for small, globular proteins such as TyrRS. In the case of hyperoside RMSD with respect to protein, it was found that the ligand RMSD ranged from 0.8 to 2.6 Å. Except for a minor fluctuation, the RMSD of hyperoside was found to remain steady throughout the simulation. The maximum ligand RMSD was recorded at 59 and 66 ns, when RMSD reached 2.59 and 2.60 Å, respectively. 

Furthermore, the flexibility of the protein system was evaluated during the simulation by computing the RMSF of individual protein amino acid residues. The higher-peaking residues correspond to loop regions (highlighted by white shade) identified by MD trajectories or N- and C-terminal areas. Low RMSF values of binding site residues show that hyperoside binding to TyrRS protein is stable. From Figure 8B, it may be observed that phyto-compound hyperoside interacted with 31 amino acids of TyrRS protein during the simulation time, which were highlighted by green vertical bars, including, Tyr36 (0.425 Å), Cys37 (0.452 Å), Gly38 (0.509 Å), Ala39 (0.534 Å), Asp40 (0.769 Å), Thr42 (1.106 Å), His47 (0.975 Å), Gly49 (1.588 Å), His50 (1.015 Å), Pro53 (0.672 Å), Phe54 (0.712 Å), Leu70 (0.486 Å), Gly72 (0.59 Å), Thr75 (0.628 Å), Gly76 (0.685 Å), Ile78 (0.726 Å), Asp80 (0.788 Å), Ser82 (1.097 Å), Lys84 (1.507 Å), Arg88 (1.551 Å), Asn124 (0.655 Å), Tyr170 (0.538 Å), Gln174 (0.528 Å), Asp177 (0.505 Å), Gln190 (0.434 Å), Val191 (0.46 Å), Gly193 (0.563 Å), Asp195 (0.687 Å), Gln196 (0.604 Å), Asn199 (0.524 Å), and Pro222 (0.662 Å).

The 2D ligand interaction diagram shows the charged negative amino acid Asp establishing a major hydrogen bond with a hydroxyl group of hyperoside. The amino acids His50, Gly38, Val191, and Thr75 interacted with hyperoside for 57%, 92%, 43%, and 57% of simulation time, respectively. Additionally, there are intramolecular hydrogen bonds in hyperoside between the hydrogen atom of hydroxyl and the carbonyl oxygen of chromen-4-one moiety (Figure 8C). The binding interactions between hyperoside and active site amino acid residues inside the binding pocket of TyrRS were computed and represented in Figure 8D. Most of the important interactions of hyperoside with the TyrRS protein determined with MD simulations are hydrogen bonds, polar (amino acids mediated hydrogen bonding) interactions, and hydrophobic interactions. 

## 4. Discussion

In the present study, we report for the first time the identification of bioactive molecules by using the ESI-MS/MS technique in the hydroalcoholic extract from *D. flabellifolia* aerial parts collected from the Saudi Arabian desert. The main identified phytoconstituents were: protocatechuic acid, chlorogenic acid, 2,5-dihydroxybenzoic acid, 4-hydroxybenzoic acid, caffeic acid, syringic acid, vanillin, sinapic acid, *p*-coumaric acid, ferulic acid, hyperoside, 2-hydroxycinnamic acid, and pinoresinol. 

As far as we know, there are few data available in the literature relating to the chemical compounds from *D. flabellifolia* extracts. In fact, Talib et al. [14] have demonstrated that *D. flabellifolia* 95% ethanolic extract collected from Wadi Hassan (Jordan) was a rich source of flavonoids and terpenoids qualitatively estimated by the thin layer chromatography technique. The same team has demonstrated the identification of several flavonoids (flavonols and flavones) by using the HPLC-MS/MS technique in *D. flabellifolia* ethanolic extract including quercetin, fisetin, kaempferol, luteolin, and apigenin. 

Our results have shown that *D. flabellifolia* hydroalcoholic extract was active against ESKAPE pathogens and *Candida* species with an increase in the mean diameter of the growth inhibition zone depending on the concentration used. The highest sensitivity for all tested microorganisms was obtained at 3 mg/disc of the tested extract. Using the schemes proposed by Gatsing et al. [31] and Moroh et al. [32], the tested extract exhibited bacteriostatic and fungistatic action against almost all tested microorganisms. No study has hitherto described the antimicrobial activity of *D. flabellifolia* extracts. While testing Jordanian *D. flabellifolia* essential oil from dried leaves, Al-Shudiefat et al. [10] reported good activity against *C. albicans* with a MIC value of about 234 µg/mL; meanwhile, for bacterial strains, the MICs values were about 234 µg/mL against *S. aureus*, 1870 µg/mL against *E. coli*, and 1872 µg/mL against *P. aeruginosa* strain. For *D. anethifolia*, Elsharkawi and colleagues [11] have demonstrated that the ethyl acetate fraction was active against different Gram-positive bacteria including *S. aureus* ATCC 25923, *S. epidermidis* ATCC 49461, *Bacillus cereus* ATCC 10876, and *S. aureus* clinical isolate tested at 500 mg/mL. The highest diameter of growth inhibition was recorded against *S. epidermidis* (14.5 ± 0.5 mm) followed by *B. subtilis* (14.0 ± 0.00 mm), while no activity was recorded against *E. coli* ATCC 35218, *K. pneumoniae* ATCC 700603, *K. pneumoniae* ATCC 27736, *P. aeruginosa*, and *A. baumannii*. 

We also reported in this study that *D. flabellifolia* hydroalcoholic extract was dominated by phenolic compounds, followed by tannins and flavonoids. The same extract showed the ability to scavenge different radicals with low IC_50_ values (IC_50_ DPPH radical = 0.014 ± 0.045 mg/mL; IC_50_ ABTS + radicals = 0.102 ± 0.024 mg/mL; and IC_50_ for the beta-carotene test = 7.80 ± 0.919 mg/mL). Previously, Mottaghipisheh et al. [13] reported that the IC_50_ value of the free radical scavenging activity of *D. anethifolia* ethanolic extract was estimated at 122.02 ppm and 354.37 ppm for the ethyl acetate extract. In addition, Elsharkawi and colleagues [11] revealed the presence of high reduction capacity (EC_50_: 0.63 ± 0.03 g/L) and ability to scavenge the free radicals of DPPH with an IC_50_ of 0.38 ± 0.02 g/L in ethyl acetate fraction of *D. anethifolia*. 

Overall, the good antimicrobial activity against all tested Gram-positive/Gram-negative and *Candida* species, and the antioxidant activity of the tested hydroalcoholic extract can be attributed to the presence of many molecules with potential biological activities (Table 6).

Molecular docking studies were performed in order to explore binding affinity and interaction of identified phyto-compounds against bacterial, fungal, and human peroxiredoxin 5 receptor. A docking score was used to determine the binding affinities of the phyto-compounds to target proteins. A lower docking score indicates a higher affinity. Luteolin 7-glucoside connected with Arg1122 and manganese (II) ion in the *S. aureus* IIA topoisomerase target via π -cationic and metal coordinates, respectively. This interaction is comparable to that of the co-crystallized standard drug ciprofloxacin. According to the study, the binding site cavity’s metal interaction had an impact on the substance’s physicochemical properties and antibacterial activity [103,104]. Free radical-scavenging activity properties using the 3-D crystallographic peroxiredoxin 5 (1HD2) were carried out to explore the identified phyto-compounds’ recognition in the active site as potential antioxidants. With a docking score of -5 kcal/mol, five phyto-compounds, namely (−)-epicatechin, eriodictyol, 2,5-dihydroxybenzoic acid, chlorogenic acid, luteolin 7-glucoside, and vanillin, showed promising antioxidant activity by appropriate orientation at the binding active site of human peroxiredoxin protein. In fungal target *C. albicans* Sap1 and bacterial *TyrRS* protein, the phyto-compound hyperoside showed the highest binding affinity. SB-219383 and its analogs are a family of bacterial TyrRS inhibitors that are both potent and selective. These inhibitors’ crystal structures have been solved in complex with the TyrRS from *S. aureus*, the bacterium that causes the majority of hospital-acquired infections, according to crystal structure. SB-219383 and its analogs interacted with charged negative amino acids Asp195 and Asp80, as well as hydrophobic Tyr170, in the active region of TyrRS from *S. aureus* [105]. Our docking results for hyperoside match those found in the deposited crystal structure, indicating that it has potential antibacterial activity by inhibiting TyrRS *S. aureus*. Because of its high affinity for microbial targets, hyperoside was selected for MD simulation studies. The RMSD values of the protein Cα atoms were used to calculate the stability of the protein–ligand complex during dynamics analysis. In fact, Cα atoms in proteins’ RMSD is a crucial parameter of the MD simulation trajectory that is used to investigate the backbone deviation of a single frame created in a dynamic environment. The unfolding of the protein molecule results in a high RMSD value, which suggests compactness. The steady fluctuation in the RMSD value across the simulation time indicates that the protein–ligand complex has equilibrated [106,107,108,109,110]. It is anticipated that the lesser RMSD value during the simulation reveals that the protein–ligand complex is more stable. In contrast, the higher RMSD value indicates the protein–ligand complex is less stable [111,112,113]. The overall RMSD revealed that fluctuations in the range of 1.6 Å to 2.8 Å were within the standard range (1–3 Å) of RMSD, indicating that the protein–ligand complex is stable. Throughout the simulation process, the RMSF value shows the mobility and flexibility of each amino acid in the protein. In this plot, the peaks indicate the fluctuation of each amino acid residue over the entire simulation. It implies that higher RMSF values represent higher residue flexibility, whereas lower RMSF values reflect less residue flexibility and better system stability. If the residues in the active site and main chain fluctuated slightly, it indicated that the conformational change was minimal, implying that the reported lead compound was firmly bound within the cavity of the target protein binding pocket [114,115]. In addition, it was observed that the phyto-compound hyperoside interacted with 31 amino acids of the TyrRS protein during the simulation period, which was highlighted by the green vertical bar. All these intercalated residues have low RMSF values, indicating that the hyperoside binding to TyrRS protein is stable.

## 5. Conclusions

In summary, we report for the first time in this study the identification of various phenolic compounds in the hydroalcoholic extract from *D. flabellifolia* aerial parts growing wild in Saudi Arabia. The tested extract possessed good antimicrobial activities and exhibited notable potency in scavenging free radicals. Docking studies on the identified phyto-compounds in the bacterial, fungal, and peroxiredoxin 5 receptors were carried out to confirm the in vitro results, and they exhibited satisfactory binding profiles. In-depth protein–ligand interaction stability in the dynamic state was evaluated using 100 ns MD simulation studies, which revealed a significant binding affinity of the discovered hyperoside towards the TyrRS protein, implying anti-bacterial effectiveness via TyrRS enzyme inhibition. The obtained results highlighted the possible use of this plant species as a source of molecules with therapeutic effects to be used as antimicrobial and antioxidant agents.

## Figures and Tables

**Table 1:** Phytochemical profiling of *D. flabellifolia* methanol/water extract by using ESI–MS/MS technique.

No	Identified Compounds	Retention Time (min)	Abondance (mg/Kg of Extract)	Chemical Formula	Molecular Weight (g/mol)
1	Gallic acid	8.891	8.78 ± 0.14	C_7_H_6_O_5_	170.12
2	Protocatechuic acid	10.818	25.69 ± 0.395	C_7_H_6_O_4_	154.12
3	3,4-Dihydroxyphenylacetic acid	11.224	1.61 ± 0.135	C_8_H_8_O_4_	168.15
4	Pyrocatechol	11.506	14.36 ± 0.315	C_6_H_6_O_2_	110.11
5	Chlorogenic acid	11.802 *	5980.96 ± 73.12	C_16_H_18_O_9_	354.31
6	2,5-Dihydroxybenzoic acid	12.412	59.74 ± 0.945	C_7_H_6_O_4_	154.12
7	4-Hydroxybenzoic acid	12.439	19.25 ± 0.98	C_7_H_6_O_3_	138.12
8	(-)-Epicatechin	12.458 *	5.15 ± 0.1	C_15_H_14_O_6_	290.27
9	Caffeic acid	12.841	70.90 ± 1.75	C_9_H_8_O_4_	180.16
10	Syringic acid	12.963	28.92 ± 0.51	C_9_H_10_O_5_	198.17
11	3-Hydroxybenzoic acid	13.259	13.30 ± 0.405	C_7_H_6_O_3_	138.12
12	Vanillin	13.379	25.03 ± 0.785	C_8_H_8_O_3_	152.15
13	Sinapic acid	13.992	61.74 ± 2.79	C_11_H_12_O_5_	224.21
14	*p*-Coumaric acid	14.022	55.11 ± 0.765	C_9_H_8_O_3_	164.16
15	Ferulic acid	14.120	180.58 ± 2.77	C_10_H_10_O_4_	194.18
16	Luteolin 7-glucoside	14.266	5.42 ± 0.5	C_21_H_20_O_11_	448.4
17	Hyperoside	14.506 *	26.55 ± 0.635	C_21_H_20_O_12_	464.4
18	Rosmarinic acid	14.600	6.05 ± 0.025	C_18_H_16_O_8_	360.3
19	Apigenin 7-glucoside	14.781 *	2.52 ± 0.25	C_21_H_20_O_10_	432.4
20	2-Hydroxycinnamic acid	15.031	31.28 ± 0.015	C_9_H_8_O_3_	164.16
21	Pinoresinol	15.118	13.13 ± 0.55	C_20_H_22_O_6_	358.4
22	Eriodictyol	15.247	2.29 ± 0.005	C_15_H_12_O_6_	288.25
23	Quercetin	15.668	2.27 ± 0.075	C_15_H_10_O_7_	302.23

*: Compounds identified by positive Ionization mode.

**Table 2:** Antioxidant activities of *D. flabellifolia* methanol/water extract compared to known drugs.

Test Systems	*D. flabellifolia* (Methanol-80%)	Butylated Hydroxytoluene	Ascorbic Acid
DPPH IC_50_ (mg/mL)	0.014 ± 0.045 ^a^	0.023 ± 3 × 10^−4 b^	0.022 ± 5 × 10^−4 b^
ABTS IC_50_ (mg/mL)	0.102 ± 0.024 ^a^	0.018 ± 4 × 10^−4 b^	0.021 ± 0.001 ^b^
β-carotene IC_50_ (mg/mL)	7.80 ± 0.919 ^a^	0.042 ± 3.5 × 10^−3 b^	0.017 ± 0.001 ^b^

The letters (a,b) indicate a significant difference between the different antioxidant methods according to the Duncan test (*p* < 0.05).

**Table 3:** Determination of MICs, MBCs, and MBC/MIC ratio values for ESKAPE strains as compared to known drugs.

Code	ESKAPE Bacterial Strains	*D. flabellifolia* Methanol-80% Extract (mg/mL)			Ampicillin (mg/mL)	
		MIC	MBC	MBC/MIC Ratio	MIC	MBC
260	*Enterococcus faecium*	25	100	=4; Bactericidal	0.625	5
445	*Staphylococcus aureus* (MRSA)	12.5	100	8; Bacteriostatic	0.625	5
259	*Staphylococcus aureus*	12.5	100	8; Bacteriostatic	0.625	1.25
140 BC	*Staphylococcus hominis*	12.5	100	8; Bacteriostatic	0.625	2.5
BC 161	*Staphylococcus epidermidis*	12.5	100	8; Bacteriostatic	0.312	0.625
147	*Klebsiella pneumoniae*	25	200	8; Bacteriostatic	0.625	5
486	*Acinetobacter baumannii*	25	200	8; Bacteriostatic	1.25	5
249	*Pseudomonas aeruginosa*	25	200	8; Bacteriostatic	2.5	5
525	*Pseudomonas aeruginosa*	25	200	8; Bacteriostatic	2.5	5
268	*Enterobacter faecalis*	12.5	100	8; Bacteriostatic	0.312	2.5
235	*Enterobacter cloacae*	50	200	=4; Bacteriostatic	0.625	1.25
215	*Escherichia coli*	25	200	8; Bacteriostatic	1.25	5

**Table 4:** Determination of MICs, MFCs, and MFC/MIC ratios for *Candida* species as compared to Amphotericin B.

Code	*Candida* sp. Strains	*D. flabellifolia* Methanol-80% Extract (mg/mL)			Amphotericin B (mg/mL)	
		MIC	MFC	MFC/MIC Ratio	MIC	MFC
A_1_	*Candida utilis* ATCC 9255	25	200	8; Fungistatic	0.78	1.56
A_8_	*Candida tropicalis* ATCC 1362	25	200	8; Fungistatic	0.195	0.78
A_4_	*Candida guillermondii* ATCC 6260	25	200	8; Fungistatic	0.097	1.56
A_15_	*Candida albicans* ATCC 20402	25	100	=4; Fungicidal	0.195	0.39

**Table 5:** Interacting active site residues of receptors with the best phyto-compounds identified in *D. flabellifolia* hydroalcoholic extract.

Name of Complex	Interacting Residues
Luteolin 7-glucoside-2XCT	Arg1112(5.54 Å): [Arg N of NH2) -Lig (Phenyl ring) *] Mn2492(1.84 Å): [ Mn-Lig (O of OH) **]
	Mn2492(1.80 Å): [ Mn-Lig (O of OH) **]
	Mn2492(1.81 Å): [ Mn-Lig (O of OH) **]
(−)-Epicatechin -1HD2	Thr44 (1.84 Å): [Thr (O of COO^−^) -Lig (H of OH) ^d^]
	Cys47 (2.76 Å): [Cys (H of NH) ^d^-Lig (O of OH)]
	Arg127 (2.70 Å): [Arg (H of NH) ^d^-Lig (O of OH)]
	Asp145 (1.70 Å): [Asp (O of COO^−^)-Lig (H of OH) ^d^]
	Thr147 (2.20 Å): [Asp (O of COO^−^)-Lig (H of OH) ^d^]
Hyperoside-2QZW	Thr6 (1.92 Å): [Thr (H of NH) ^d^ -Lig (O of OH)]
	Asp17 (1.74 Å): [Asp (O of COO^−^)-Lig (H of OH) ^d^]
	Asp17 (1.84 Å): [Asp (O of COO^−^)-Lig (H of OH) ^d^]
	Asp17 (1.92 Å): [Asp (O of NHCO^−^)-Lig (H of OH) ^d^]
	Lys26 (1.80 Å): [Lys (H of NH) ^d^ -Lig (O of OH)]
	Gly103 (2.62 Å): [Gly H of NH) ^d^ -Lig (O of C-O-C)]
	Ala104 (2.18 Å): [Ala O of COO^−^) -Lig (H of OH) ^d^]
Hyperoside-1JIJ	Gly38 (2.70 Å): [Gly (H of NH) ^d^ -Lig (O of C = O)]
	Asp80(1.85 Å): [Asp (O of COO^−^)-Lig (H of OH) ^d^]
	Lys84 (1.91 Å): [Lys (H of NH) ^d^ -Lig (O of OH)]
	Asp177 (1.85 Å): [Asp (H of NH) ^d^ -Lig (O of OH)]
	Gly193 (2.28 Å): [Gly (H of NH) ^d^ -Lig (O of OH)]
	Asp195 (1.93 Å): [Asp (O of COO^−^)-Lig (H of OH) ^d^]

^d^ Hydrogen bond donor; * π-Cation interaction; ** Metal interaction.

**Table 6:** Literature survey showing some biological activities of the identified molecules in *D. flabellifolia* hydroalcoholic extract.

Identified Compounds	Biological Activities	References
Gallic acid	Antioxidant, antimicrobial, anticancer, anti-inflammatory, gastroprotective, cardioprotective, neuroprotective, anti-hyperlipidemia, anti-obesity, and anti-diabetes.	[51,52,53]
Protocatechuic acid	Antiviral, anti-oxidation, antibacterial, anti-apoptotic, anti-inflammatory, anti-atherosclerotic, antioxidant, and neuroprotective.	[54,55,56,57,58]
3,4-Dihydroxyphenylacetic acid	Decrease in the formation of amyloid fibrils and modulator of cell fate in Parkinson’s disease.	[59,60]
Pyrocatechol	Antibacterial, antitumor, antioxidant and cytotoxic activities.	[61]
Chlorogenic acid	Anti-metastatic, anti-oxidative, nephroprotective, anti-inflammatory, anti-diabetic, anti-hypertensive, hepatoprotective, anti-bacterial, neuroprotective, anti-proliferative, central nervous system stimulator, anti-obesity, cardioprotective, anti-pyretic, anti-viral, anti-angiogenic.	[62]
2,5-Dihydroxybenzoic acid	Anti-inflammatory, anti-oxidant, antibacterial, muscle relaxant, anticarcinogenic, nephroprotective, hepatoprotective, cardioprotective, neuroprotective.	[63,64]
4-Hydroxybenzoic acid	Antibacterial, antiviral, antisickling agent, antialgal, antimutagenic, estrogenic agent, anti-inflammatory, antioxidant.	[65]
(−)-Epicatechin	Antiviral (Anti-SARS-CoV-2 virus), gastroprotective, cardioprotective, neuroprotective, hepatoprotective, antioxidant, anti-inflammatory, antidiabetic.	[66,67,68]
Caffeic acid	Anti-inflammatory, anticancer, antiviral, antioxidant, antihyperglycemic, antidepressive, antibacterial.	[69,70,71]
Syringic acid	Anti-oxidant, anti-microbial, anti-inflammation, antiangiogenic, anti-cancer, anti-diabetic, hepatoprotective, cardioprotective, neuroprotective.	[72]
3-Hydroxybenzoic acid	Antifungal, antimutagenic, antisickling, estrogenic, antimicrobial.	[73]
Vanillin	Anticancer, neuroprotective, antihyperglycemic, anti-hyperlipidemic, anti-inflammatory, antimicrobial, antioxidant, antisickling, cardioprotective.	[74,75,76]
Sinapic acid	Antioxidant, anti-inflammatory, anti-cancer, antihypertensive, cardioprotective, neuroprotective, renoprotective, hepatoprotective, anti-hyperglycemic, anti-diabetic.	[77,78]
*p*-Coumaric acid	Antioxidant, anti-inflammatory, anti-platelet aggregation, analgesic, anticancer, neuroprotective, anti-necrotic, anti-cholestatic, anti-amoebic.	[79,80]
Ferulic acid	Anti-inflammatory, antioxidant, antimicrobial activity, anticancer, and antidiabetic	[81,82,83]
Luteolin 7-glucoside	Antioxidant, anti-inflammatory, antiaging, anticancer, vasoprotective	[84,85]
Hyperoside	Anti-inflammatory, anti-thrombotic, antidiabetic, anti-viral, anti-fungal, hepato-protective, antioxidant, neuroprotective, antidepressant, cardioprotective, antidiabetic, anticancer, hepatoprotective, Immuno-modulatory activity.	[86,87,88]
Rosmarinic acid	Cytoprotective, antioxidative, antibacterial, antiviral, astringent, analgesic, anti-inflammatory, antihyperglycemic, hepatoprotective, immunomodulatory, anticancer, cardioprotective, neuroprotective.	[89,90,91]
Apigenin 7-glucoside	Anti-inflammatory, anticandidal, anticancer, antiviral, antibacterial, antioxidant, pro-apoptotic, antimutagenic, antiproliferative, antiallergic, inhibits xenobiotic-metabolizing enzymes.	[92,93,94]
2-Hydroxycinnamic acid	Inhibition of HIV/SARS-CoV S pseudovirus.	[95]
Pinoresinol	Neuroprotective, vasorelaxant, hepatoprotective, anti-inflammatory, anticancer.	[96,97,98]
Eriodictyol	Cardioprotective, skin protection, antitumor, neuroprotective, antioxidant, antidiabetic, anti-inflammatory, cytoprotective, hepatoprotective, analgesic.	[99,100]
Quercetin	Anticancer, antiviral, antiprotozoal, antimicrobial, anti-allergy, anti-inflammatory, cardioprotective, sedative, immunostimulant.	[101,102]

## Data Availability

The data presented in this study are available in the article and supplementary materials.

## Supplementary Materials

The following supporting information can be downloaded at: https://www.mdpi.com/article/10.3390/antiox11112174/s1, Supplementary material (S1). ESI–MS/MS Parameters and analytical characteristics for the Analysis of Target Analytes by MRM Negative and Positive Ionization Mode.; Supplementary material (S2). Calibration curves and sensitivity properties of the method; Supplementary material (S3). LC–ESI–MS/MS MRM chromatograms of phenolic compounds. 1–31 represent the chromatograms of gallic acid, protocatechuic acid, 3,4-dihydroxyphenylacetic acid, chlorogenic acid, (−)-epicatechin, caffeic acid, 3-hydroxybenzoic acid, vanillin, verbascoside, taxifolin, p-coumaric acid, luteolin 7-glucoside, hyperoside, rosmarinic acid, apigenin 7-glucoside, 2-hydroxycinnamic acid, eriodictyol, quercetin, luteolin, apigenin, (+)-catechin, pyrocatechol, 2,5-dihydroxybenzoic acid, 4-hydroxybenzoic acid, vanillic acid, syringic acid, sinapic acid, ferulic acid, hesperidin, pinoresinol and kaempferol, respectively; Supplementary material (S4). LC–ESI–MS/MS MRM chromatogram of D. flabellifolia methanol/water extract; Supplementary material (S5). Result of the docking experiment performed between the Target proteins and the identified phyto-compounds.
