# Supplementary material for: Phytochemical Analysis, Antioxidant, and Antimicrobial Activities of Ducrosia flabellifolia: A Combined Experimental and Computational Approaches
Source: Antioxidants (Basel). 2022 Nov 2;11(11):2174. doi: 10.3390/antiox11112174 (PMC9686979; doi:10.3390/antiox11112174)
Supplement: Supplementary file 1 [file antioxidants-11-02174-s001.zip › antioxidants-1959368-supplementary.pdf]

Supplementary material (S1). ESI–MS/MS Parameters and analytical characteristics for the Analysis of Target Analytes by MRM Negative and Positive Ionization Mode.

Target compounds	Rt (min)	Precursor ion	MRM1 (CE, V)	MRM2 (CE, V)
Compounds analyzed by NI mode				
Gallic acid	8.891	168.9 [M − H]−	125.0 (10)	–
Protocatechuic acid	10.818	152.9 [M − H]−	108.9 (12)	–
3,4-Dihydroxyphenylacetic acid	11.224	167.0 [M − H]−	123.0 (2)	–
(+)-Catechin	11.369	289.0 [M − H]−	245.0 (6)	202.9 (12)
Pyrocatechol	11.506	109.0 [M − H]−	90.6 (18)	52.9 (16)
2,5-Dihydroxybenzoic acid	12.412	152.9 [M − H]−	109.0 (10)	–
4-Hydroxybenzoic acid	12.439	136.9 [M − H]−	93.1 (14)	–
Caffeic acid	12.841	179.0 [M − H]−	135.0 (12)	–
Vanillic acid	12.843	166.9 [M − H]−	151.8 (10)	122.6 (6)
Syringic acid	12.963	196.9 [M − H]−	181.9 (8)	152.8 (6)
3-Hydroxybenzoic acid	13.259	137.0 [M − H]−	93.0 (6)	–
Vanillin	13.397	151.0 [M − H]−	136.0 (10)	–
Verbascoside	13.589	623.0 [M − H]−	461.0 (26)	160.8 (36)
Taxifolin	13.909	303.0 [M − H]−	285.1 (2)	125.0 (14)
Sinapic acid	13.992	222.9 [M − H]−	207.9 (6)	163.8 (6)
p-Coumaric acid	14.022	162.9 [M − H]−	119.0 (12)	–
Ferulic acid	14.120	193.0 [M − H]−	177.8 (8)	134.0 (12)
Luteolin 7-glucoside	14.266	447.1 [M − H]−	285.0 (24)	–
Rosmarinic acid	14.600	359.0 [M − H]−	196.9 (10)	160.9 (10)
2-Hydroxycinnamic acid	15.031	162.9 [M − H]−	119.1 (10)	–
Pinoresinol	15.118	357.0 [M − H]−	151.0 (12)	135.7 (34)
Eriodictyol	15.247	287.0 [M − H]−	151.0 (4)	134.9 (22)
Quercetin	15.668	301.0 [M − H]−	178.6 (10)	151.0 (16)
Kaempferol	16.236	285.0 [M − H]−	242.8 (16)	229.1 (18)
Compounds analyzed by PI mode				
Chlorogenic acid	11.802	355.0 [M + H]+	163.0 (10)	–
(−)-Epicatechin	12.458	291.0 [M + H]+	139.1 (12)	122.9 (36)
Hesperidin	14.412	611.1 [M + H]+	449.2 (4)	303.0 (20)
Hyperoside	14.506	465.1 [M + H]+	303.1 (8)	–
Apigenin 7-glucoside	14.781	433.1 [M + H]+	271.0 (18)	–
Luteolin	15.923	287.0 [M + H]+	153.1 (34)	135.1 (36)
Apigenin	16.382	271.0 [M + H]+	153.0 (34)	119.1 (36)

R_t, retention time; NI, negative ion; and PI, positive ion.

Supplementary material (S2). Calibration curves and sensitivity properties of the method.

Compounds	Linearity and sensitivity characteristics				
	Range (μg/L)	Linear equation	R^2	LOD (μg/L)	LOQ (μg/L)
Gallic acid	5–500	y = 4.92x − 14.36	0.9988	1.39	4.63
Protocatechuic acid	2.5–500	y = 5.92x – 8.55	0.9990	1.15	3.83
3,4-Dihydroxyphenylacetic acid	5–500	y = 4.99x − 11.12	0.9990	1.31	4.37
(+)-Catechin	10–500	y = 1.54x + 1.66	0.9974	3.85	12.83
Pyrocatechol	25–400	y = 0.13x − 0.44	0.9916	9.43	31.43
Chlorogenic acid	1–500	y = 11.18x + 23.24	0.9995	0.58	1.93
2,5-Dihydroxybenzoic acid	5–500	y = 3.85x − 12.86	0.9980	2.23	7.43
4-Hydroxybenzoic acid	5–500	y = 7.02x + 18.95	0.9996	1.69	5.63
(−)-Epicatechin	5–500	y = 10.21x – 8.25	0.9971	1.88	6.27
Caffeic acid	5–500	y = 12.88x + 17.02	0.9997	3.16	10.53
Vanillic acid	10–500	y = 0.51x − 1.72	0.9968	2.54	8.47
Syringic acid	10–500	y = 0.79x − 1.39	0.9975	3.82	12.73
3-Hydroxybenzoic acid	5–500	y = 3.82x – 13.04	0.9991	1.69	5.63
Vanillin	50–500	y = 2.23x + 112.65	0.9926	14.98	49.93
Verbascoside	2.5–500	y = 8.40x − 24.12	0.9988	0.85	2.83
Taxifolin	5–500	y = 11.44x + 10.21	0.9993	1.91	6.37
Sinapic acid	5–500	y = 2.02x − 6.10	0.9974	2.51	8.37
p-Coumaric acid	5–500	y = 16.89x + 43.52	0.9997	1.88	6.27
Ferulic acid	5–500	y = 3.54x − 3.38	0.9992	1.46	4.87
Luteolin 7-glucoside	1–500	y = 43.36x + 145.44	0.9996	0.47	1.57
Hesperidin	5–500	y = 6.03x + 0.38	0.9993	1.81	6.03
Hyperoside	2.5–500	y = 16.01x − 1.84	0.9998	1.01	3.37
Rosmarinic acid	1–500	y = 9.72x − 16.08	0.9989	0.52	1.73
Apigenin 7-glucoside	1–500	y = 22.15x − 29.10	0.9983	0.43	1.43
2-Hydroxycinnamic acid	1–500	y = 16.72x − 26.94	0.9996	0.68	2.27
Pinoresinol	10–500	y = 0.78x − 2.86	0.9966	3.79	12.63
Eriodictyol	2.5–500	y = 13.75x – 1.02	0.9998	0.83	2.77
Quercetin	5–500	y = 15.55x – 17.12	0.9997	1.28	4.27
Luteolin	5–500	y = 8.48x + 24.25	0.9992	1.39	4.63
Kaempferol	10–500	y = 0.78x – 2.88	0.9959	3.25	10.83
Apigenin	2.5–500	y = 10.88x + 24.36	0.9987	0.94	3.13

LOD and LOQ: limit of detection and limit of quantification, respectively.

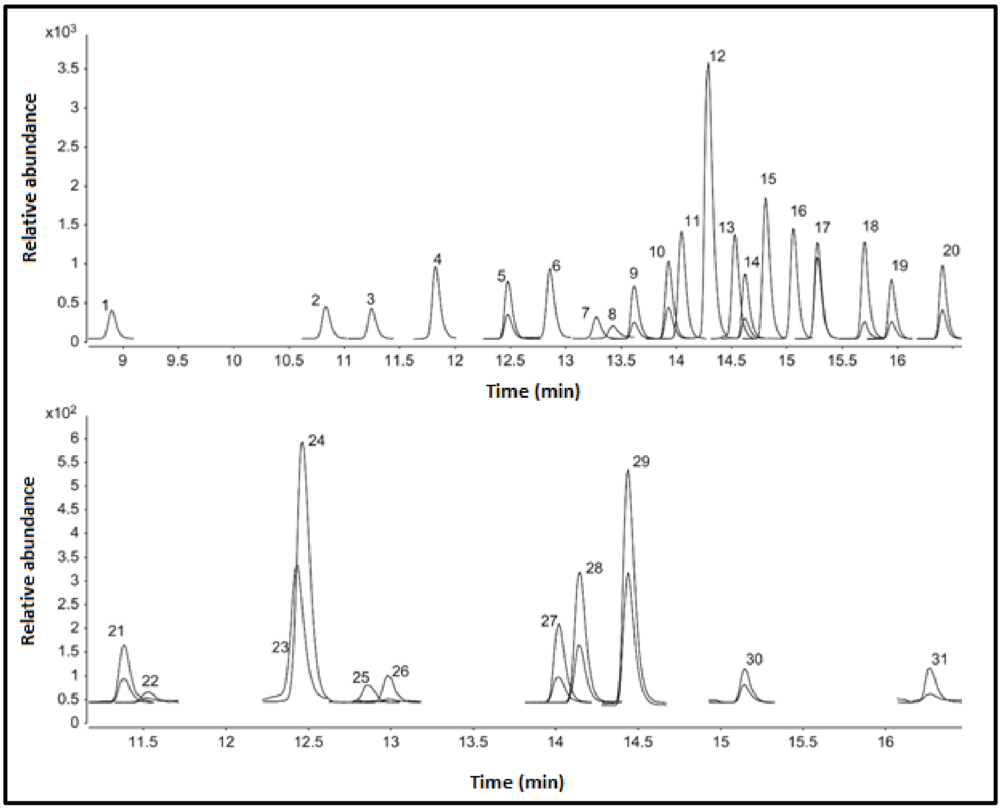

Supplementary material (S3). LC–ESI–MS/MS MRM chromatograms of phenolic compounds. 1–31 represent the chromatograms of gallic acid, protocatechuic acid, 3,4-dihydroxyphenylacetic acid, chlorogenic acid, (−)-epicatechin, caffeic acid, 3-hydroxybenzoic acid, vanillin, verbascoside, taxifolin, p-coumaric acid, luteolin 7-glucoside, hyperoside, rosmarinic acid, apigenin 7-glucoside, 2-hydroxycinnamic acid, eriodictyol, quercetin, luteolin, apigenin, (+)-catechin, pyrocatechol, 2,5-dihydroxybenzoic acid, 4-hydroxybenzoic acid, vanillic acid, syringic acid, sinapic acid, ferulic acid, hesperidin, pinoresinol and kaempferol, respectively.

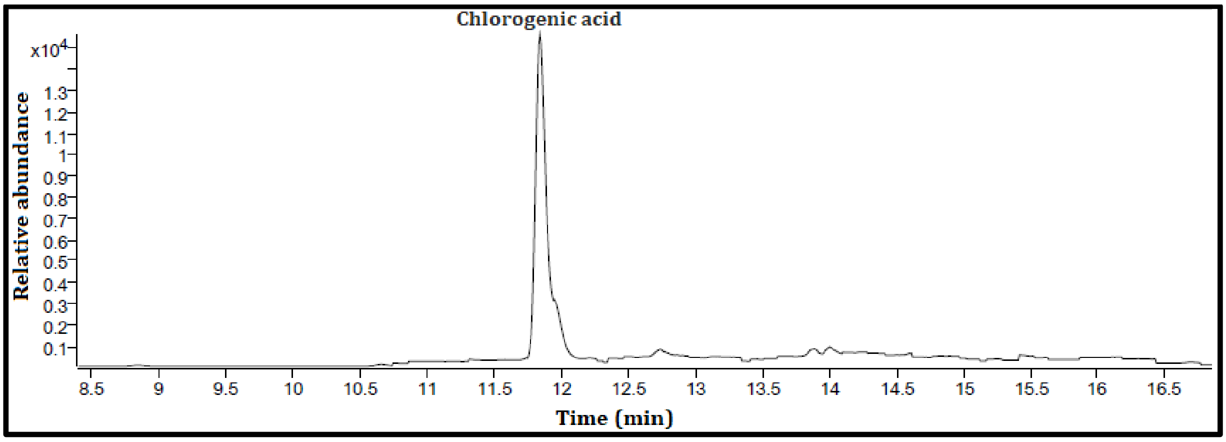

Supplementary material (S4). LC–ESI–MS/MS MRM chromatogram of *D. flabellifolia* methanol/water extract.

Supplementary material (S5). Result of the docking experiment performed between the Target proteins and the identified phyto-compounds.

N^{O}	Identified compounds	Protein Targets			
		2XCT	1HD2	2QZW	1JIJ
1	Gallic acid	-5.802	-4.844	-4.413	-6.881
2	Protocatechuic acid	-5.523	-4.464	-4.303	-6.617
3	3,4-Dihydroxyphenylacetic acid	-5.432	-4.574	-4.207	-6.763
4	Pyrocatechol	-5.163	-4.761	-4.274	-5.554
5	Chlorogenic acid	-7.899	-5.11	-4.954	-8.695
6	2,5-Dihydroxybenzoic acid	-5.668	-5.249	-4.171	-6.276
7	4-Hydroxybenzoic acid	-5.011	-4.564	-4.194	-5.57
8	(-)-Epicatechin	-9.836	-5.791	-5.461	-7.731
9	Caffeic acid	-6.34	-4.407	-4.753	-6.243
10	Syringic acid	-5.512	-4.146	-5.042	-6.861
11	3-Hydroxybenzoic acid	-5.181	-5.001	-4.393	-6.25
12	Vanillin	-5.708	-5.02	-4.947	-5.96
13	Sinapic acid	-5.677	-3.687	-4.084	-6.381
14	p-Coumaric acid	-5.965	-4.562	-5.162	-6.325
15	Ferulic acid	-5.808	-4.462	-4.126	-6.094
16	Luteolin 7-glucoside	-12.562	-5.06	-4.344	-8.212
17	Hyperoside	-9.159	-4.813	-6.055	-8.852
18	Rosmarinic acid	-8.37	-4.205	-4.531	-7.58
19	Apigenin 7-glucoside	-12.514	-4.717	-4.785	-7.717
20	2-Hydroxycinnamic acid	-7.901	-3.859	-4.032	-5.431
21	Pinoresinol	-7.654	-4.269	-4.792	-6.019
22	Eriodictyol	-9.582	-5.255	-5.814	-7.296
23	Quercetin	-10.06	-4.976	-4.515	-7.211
